# Bio-Inspired Drug Delivery Systems: From Synthetic Polypeptide Vesicles to Outer Membrane Vesicles

**DOI:** 10.3390/pharmaceutics15020368

**Published:** 2023-01-21

**Authors:** Yu Zhang, Yiming Lu, Yixin Xu, Zunkang Zhou, Yichong Li, Wei Ling, Wenliang Song

**Affiliations:** 1 School of Pharmacy, Shanghai University of Medicine & Health Sciences, Shanghai 201318, China; 2 School of Materials and Chemistry, University of Shanghai for Science and Technology, Shanghai 200093, China

**Keywords:** drug delivery, polypeptides vesicles, outer membrane vesicles, biological properties

## Abstract

Nanomedicine is a broad field that focuses on the development of nanocarriers to deliver specific drugs to targeted sites. A synthetic polypeptide is a kind of biomaterial composed of repeating amino acid units that are linked by peptide bonds. The multiplied amphiphilicity segment of the polypeptide could assemble to form polypeptide vesicles (PVs) under suitable conditions. Different from polypeptide vesicles, outer membrane vesicles (OMVs) are spherical buds of the outer membrane filled with periplasmic content, which commonly originate from Gram-negative bacteria. Owing to their biodegradability and excellent biocompatibility, both PVs and OMVs have been utilized as carriers in delivering drugs. In this review, we discuss the recent drug delivery research based on PVs and OMVs. These related topics are presented: (1) a brief introduction to the production methods for PVs and OMVs; (2) a thorough explanation of PV- and OMV-related applications in drug delivery including the vesicle design and biological assessment; (3) finally, we conclude with a discussion on perspectives and future challenges related to the drug delivery systems of PVs and OMVs.

## 1. Introduction

Pharmacologically active agents (also known as therapeutics or drugs) are used in medicine to control or stop the progression of disease [1,2]. Improved delivery methods and a better understanding of how drug administration affects safety and efficacy have been thoroughly discussed [3,4]. Drug delivery systems (DDSs) are technological systems that comprehensively regulate the distribution of drugs in the organism in terms of space, time, and dose [5,6,7,8,9,10]. The goal is to deliver the right amount of drug to the right place at the right time, thus increasing the efficiency of drug utilization, improving efficacy, reducing costs, and reducing toxic side effects [11,12]. To date, DDS usage has seen widespread integration in synthetic chemistry, materials science, biology, and medical sciences and is becoming increasingly prevalent in clinical practice. However, drug delivery targeting is not easily controlled because the release rate, the targeted sites, and the stability of the carrier and drug are inconsistent in different environments. Understanding the drug delivery characteristics of specific vesicles is a significant basis for their future application.

Polypeptides are crucial, artificially prepared biomaterials [13,14]. Their outstanding biocompatibility, appropriate biodegradability, and biological chain design abilities allow polypeptides to be employed in a variety of applications, such as drug delivery, antibacterial applications, and gene delivery [14,15]. In addition, unique secondary structures of polypeptides, such as α-helices and β-sheets, endow them with interesting self-assembly behavior and biological properties. Generally, three approaches can be applied to the synthesis of polypeptide materials, including solid-phase synthesis, α-amino acid N-carboxyanhydride (NCA) polymerization, and microbial fermentation [16]. Among these, the ring-opening polymerization (ROP) of NCAs represents a facile route to obtain high-molecular-weight polymers with the desired functionality. Due to the variety of ROP chemical and synthetic strategies, a polypeptide not only can be conjugated with multiple polypeptide segments, but it can also be blocked with non-peptidic materials. The peptides mentioned in this review are mainly synthetic polypeptides, which are biomaterials composed of repeating amino acid units linked by peptide bonds. In particular, amphiphilic polypeptides, which are composed of hydrophobic and hydrophilic chains, can easily self-assemble into micelles under suitable conditions. The vesicle is the most interesting assembled morphology of micelles. Further, the designed pH-responsive residues could be used as candidates to load hydrophilic cargos and be released upon appropriate stimulation. A subtle tactic to reduce unintended toxicity and increase effectiveness is the design of smart chemistry methods for programmed release in DDSs. Polymers with a variety of specific functionalities that react to internal or external stimuli and cause payloads to be released at the target site are needed. As a result, controlled release using vesicles, which is otherwise challenging to achieve in delivery systems based on other biopolymers, can be made possible by tuning the built-in molecular structures of polypeptide segments.

Different from artificially assembled polypeptide vesicles, vesicles derived from the outer membrane of Gram-negative bacteria are called OMVs [17,18,19,20]. OMVs are widely produced, vary in size and composition, and contain soluble periplasmic content. Given the diversity of OMV-producing pathways, species-dependent differences in the envelope architecture, and environmental influences on envelope composition, it is difficult to identify a single molecular or genetic basis for OMV production. OMVs were first reported more than 50 years ago in research on bacterial growth and were described to be able to release vesicles with diameters of 20 to 300 nm [21]. Since the outer membrane of the original bacteria was studied, several bacteria have been reported to be capable of producing OMVs, such as *Pseudomonas aeruginosa*, *Helicobacter pylori*, *Escherichia coli*, *Shigella* sp., *Borrelia burgdorferi*, *Vibrio* sp., *Neisseria* sp., etc. [22,23,24,25,26]. As they are derived from the membrane, OMVs mainly contain phospholipids, lipopolysaccharides, outer membrane proteins, and periplasm, along with different cytoplasmic components. These vesicles have been connected to several physiological activities because of their composition, including protein transport, nutrition acquisition, cell-to-cell communication, antibacterial activity, toxin delivery, and host immune response regulation. In addition, differently from the clear ROP polymerization and self-assembly mechanism, the mechanism of OMVs remains a mystery. Recently, owing to some gene and biochemistry studies, some clues about this complex process have been reported. In recent years, it has been found that OMVs could be used in antigens for vaccines, drug delivery, bioimaging, nanogenerators, and new adjuvants. Studies have also revealed that OMVs have great potential in the transport of small-molecule drugs. Further, it has been demonstrated how to electroporate medications into the OMV lumen. Finally, passive diffusion can be employed for tiny molecules that are generally hydrophobic and positively charged, so that they can easily interact with lipophilic membranes (Table 1).

**Figure 1:**
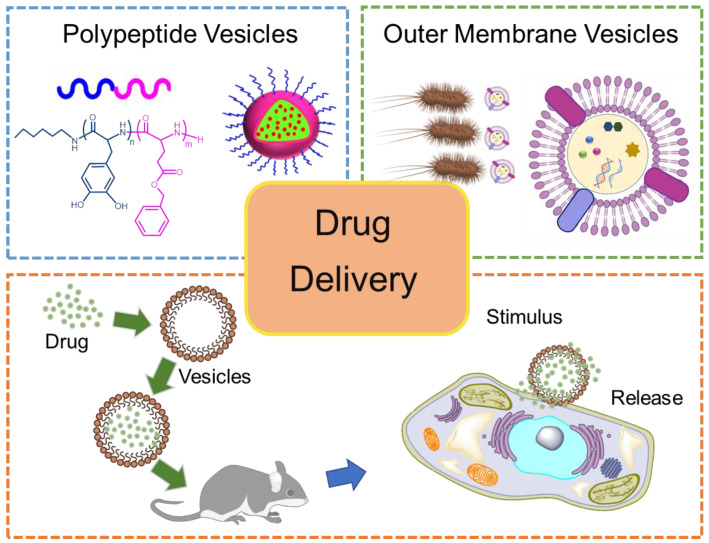
Schematic illustration of PVs and OMVs, along with their drug delivery properties.

In this review, first, we highlight recent achievements in research on artificial PVs and natural OMVs (Figure 1). The production methods of PVs and OMVs are summarized. Then, the comparison of the fabrication of PV- and OMV-based DDSs and especially their biological evaluation are extensively discussed. In the end, the current challenges regarding PVs and OMVs are also presented.

## 2. Production and Characterization of PVs and OMVs

### 2.1. Production of PVs

In polypeptide clinical transformation, the precise control of chemical structures and the polydispersity index (PDI) is very important for polymer vesicle formation. In recent decades, continued efforts have been made to produce polypeptides with controlled molecular weight, narrow PDI, peptide segments, and physical–chemical biological effects [13,29]. However, solid-phase peptide synthesis and microbial fermentation have been characterized by low yield, high costs, and limited achievable chain length. Herein, we mainly focus on the polymerization method for obtaining polypeptides via the ROP of α-amino acid NCAs and the vesicles subsequently assembled in solution.

**Figure 2:**
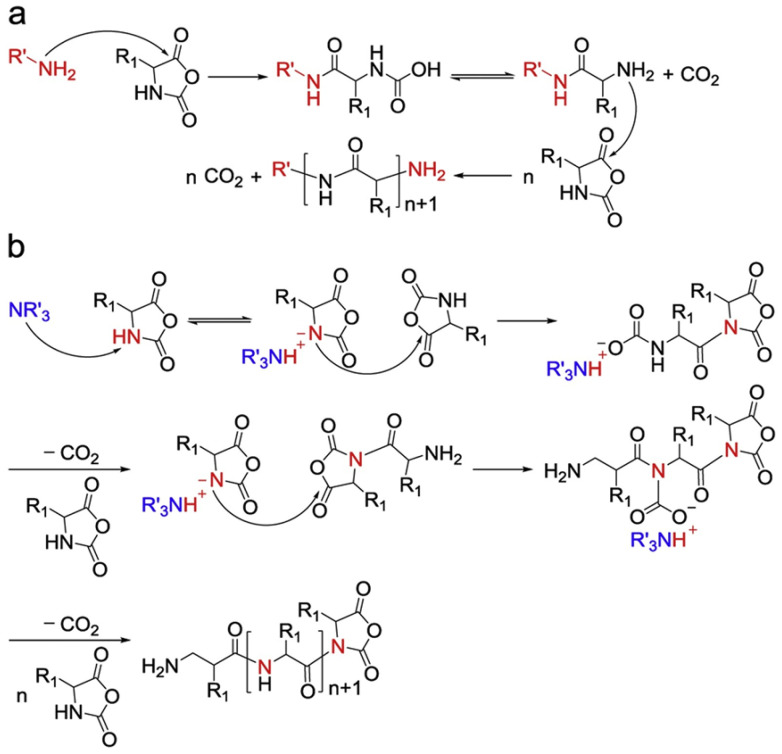
Schematic illustration of polypeptide formation mechanism, (**a**) normal amine mechanism and (**b**) active monomer mechanism. Reprinted with permission from Ref. [32]. Copyright: 1997, John Wiley & Sons, Inc.

In 1921, Curtius, Wessel, and their coworkers used NCAs to produce polypeptides using ROP methods [30]. The “normal amine mechanism” (NAM) (Figure 2a) and the “active monomer mechanism” (AMM) (Figure 2b) are the two typical routes via which nucleophiles or bases initiate the ROP of NCAs [31]. The NAM not only includes primary and secondary amines but also alcohols and thiols as initiators. Initiation often occurs due to the nucleophilic attack of the carbonyl group of the NCA ring. The intermediate product, carbamic acid, is subsequently formed due to proton transfer and ring opening. Further, the decarboxylation of carbamic acid eliminates CO_2_ and promotes the propagation of the polymer chain. It has been reported that the NAM can control the molecular weight of polypeptides and the adjustable end groups well. However, due to the side reaction that often occurs along with this process, cyclic polymers and terminated active end groups derived from the impurities in the reactions (water, reaction solvent, or contaminants in NCAs) can be found in the resulting polymers. On the other hand, the AMM mainly involves tertiary amines and alkoxides. It might occur that the resultant NCA anions catch the proton from the nitrogen group of NCAs (3-N). NCA anions can initiate further reactions with other NCAs, as propagation could further involve the attacking of the 5-CO group of other NCAs. In this case, polymerization is untraceable, generating ill-defined polypeptides [32]. It is worth pointing out that these two mechanisms often co-exist in one polymerization process, so the precise control of the reaction conditions is the main issue that needs addressing.

By considering this reported mechanism, various polymerization methods with different initiators, catalysts, and pre-designed NCAs have been reported for polypeptide design over the years. Living polymer methods can greatly enhance the speed of NCA polymerization, and the reaction time can even be minimized to a few minutes. Moreover, “Click chemistry” can be used for the post-conjugation of various groups on the polypeptide chain, thus giving the resulting polypeptide unique stimulus responsiveness. In this method, firstly, various initiators can be used, such as metal-based complexes, amine salts, silane derivatives, etc. Even without any initiators, photo-based chemistry can lead to a polypeptide with tunable molecular weight. Secondly, the catalyst has an important role in controlling the ROP of NCAs. Metallic catalysts and organic catalysts are the two main types of catalysts for controlling the speed of reaction by shortening the propagation time. Metal-based catalysts often result in metal residues on the resulting polypeptide. As organic catalysts do not have this problem, leaving no traces of metal, they offer the most freedom to control the fabrication of polymers that are suitable for various applications, especially in the biomedical area. Additionally, the conditions of high vacuum, low reaction temperatures, N_2_ flow, and UV can all be applied to polypeptide synthesis and have the ability to accelerate the reaction speed and reduce the side reaction. These improved polymerization technologies are significant, as they give researchers the chance to obtain polypeptide-based polymers with different topologies and interesting structures. Not only can linear, hinged, cyclic, and branched polypeptides be quickly obtained, but also random, alternating, block, and grafted copolypeptides can be obtained by applying sophisticated chemical synthesis skills. These copolymerized polypeptides with different topologies also greatly improve vesicle formation. 

**Figure 3:**
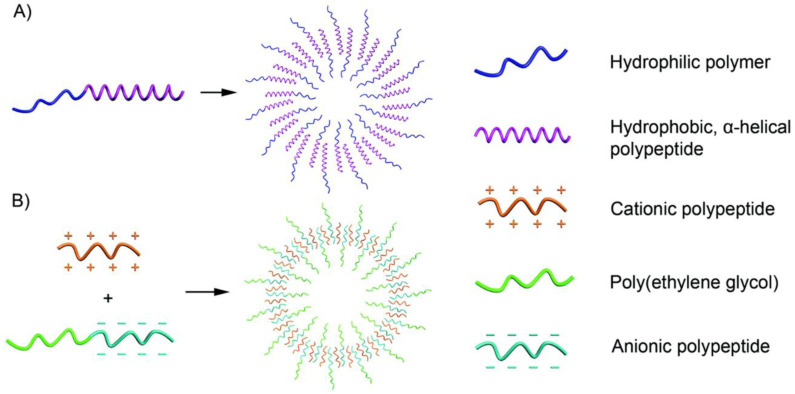
Schematic illustration of polypeptide vesicle formation. (**A**) a-helical polypeptide segments and (**B**) PVs from two polymers containing charged polypeptide segments with opposite charges. Reprinted with permission from Ref. [14]. Copyright: 2017, The Royal Society of Chemistry.

PVs are hollow spheres in shape with a bilayer wall of polymers. Commonly, they are formed through the self-assembly of amphiphilic polymers (Figure 3). These vesicles have various advantages over common polymer micelles: (1) the inner aqueous cavity can be loaded with hydrophilic cargo; (2) hydrophobic cargo can be loaded on the membrane of the vesicles; and (3) vesicles can easily disassemble to achieve suitable drug release properties. Thus, stimulus responsiveness is very important in PV formation; for instance, PV nano-assemblies can disassemble or reassemble under the simulation of pH, ROS, enzymes, or infrared light. Thus, in drug delivery, nano-assemblies can easily carry the drug compound to suitable sites and release it with the disassembly of the vesicles.

#### 2.1.1. Anisotropic Packing of α-Helical, Hydrophobic Polypeptides

The α-helical conformation can be observed in a variety of polypeptides with rigid rods, such as polyleucine and polyphenylalanine. This is attributed to the hydrophobic polypeptide segment, even at a high hydrophilic-to-hydrophobic ratio. Moreover, research also shows that randomly coiled copolymers can only form micelles, even with finely controlled components. The hydrophobic block becomes the essential component for assembling hydrophobic polypeptides (Figure 3A). For example, as early as 2005, Timothy J. Deming already reported on the charged polypeptides of poly(l-lysine)-b-poly(l-leucine) block copolypeptides, which could be assembled into vesicular assemblies [33]. Afterwards, cationic polypeptides and anionic polypeptides were both reported to be able to form vesicles under suitable conditions [34,35,36]. Polypeptides with carbohydrate moieties can be covalently linked to the side chains of amino acids and form glycopeptides, with this segment as the hydrophilic chain, and polypeptide vesicles can also be formed. Andreas Heise et al. showed that poly(γ-benzyl-l-glutamate) (PBLG)-b-poly (galactosylated propargylglycine) can also form stable vesicles, in which hydrophobic PBLG plays a decisive role [37]. Since hydrophobic polypeptide segments are limited, the hydrophilic chain can be varied. Not only peptide materials but also biodegradable polymers, such as polyethylene glycol (PEG) and hyaluronan (HYA), have been reported in research studies on vesicle preparation.

#### 2.1.2. Oppositely Charged Polymers

Amphiphilic polypeptides can be used to form vesicles using the traditional self-assembly methods introduced by Eisenberg [38] (Figure 3B). First, a solvent dissolves the amphiphilic polypeptides; after that, a solvent specific to one segment is added to the system to induce the formation of vesicles. On the other hand, Kataoka’s group further showed that oppositely charged polypeptides can be assembled into vesicles in aqueous solution. Even without any trace of organic solvent, water can induce vesicle formation. 

#### 2.1.3. Others

Recently, reports have also shown that poly(ε-caprolactone)-block-poly(lysine-stat-phenylalanine) [PCL-b-P(Lys-stat-Phe)] and poly(ε-caprolactone)-block-poly(tryptophan)-block-poly(lysine-stat-phenylalanine) might also be able to assemble into vesicles, where PCLs are the resulting hydrophobic membranes [39,40]. Specifically, these are dual corona vesicles, which are induced using solvent-switch methods. In addition, poly (L-glutamic acid) have good solubility at neutral pH; in fact, it was reported that poly (trimethylene carbonate)-b-poly(l-glutamic acid) materials could also assemble into vesicles with altered sizes of 60 nm and 130 nm at pH values of 7.4 and 10.5, respectively [41].

### 2.2. Production of OMVs

**Figure 4:**
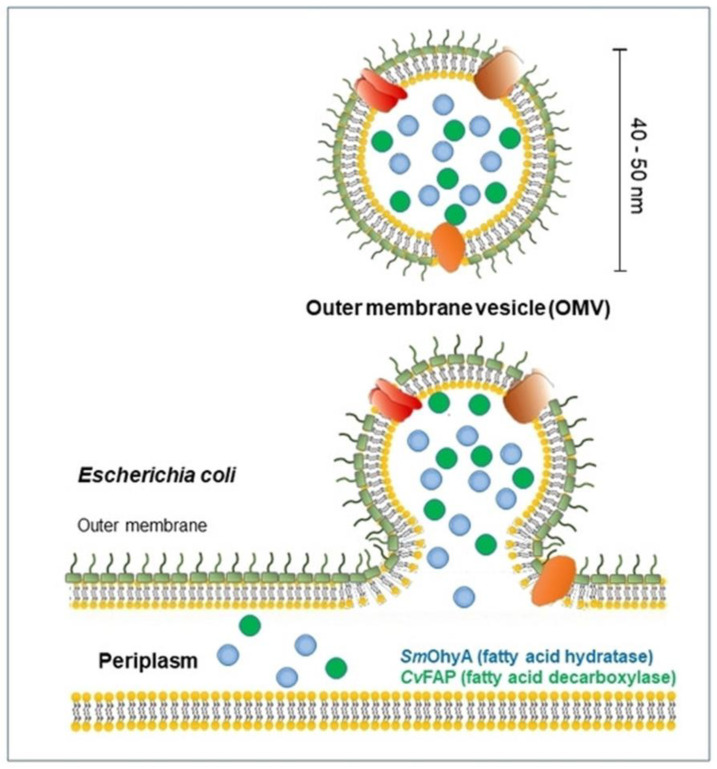
Biogenesis of OMV production in bacteria. Reprinted with permission from Ref. [44]. Copyright: 2021, John Wiley & Sons, Inc.

Cells create and release membrane-bound material, which is sometimes referred to as membrane vesicles, microvesicles, exosomes, tolerasomes, agrosomes, and virus-like particles, in all domains of life (Figure 4). The first time OMVs were observed dates to the 1960s, when Chatterjee and Das studied the cell-wall structures of *Vibrio cholera* (*V. cholera*) in vitro. Moreover, K. W. Knox et al. firstly found changes in the lysine-limited culture of Escherichia coli, characterized by cell growth with surrounding “globules” and “hollow spheres” [42]. Recently, the biosynthesis field has achieved great advances, and the physiological roles of OMVs have also been found. Moreover, related reports have shown that released vesicles contain lipids, proteins, DNA, RNA, and lipopolysaccharides [43]. In general, OMVs are vesicle structures ranging from about 20 to 300 nm and are produced by almost all Gram-negative bacteria, though a similar phenomenon has also been detected in Gram-positive bacteria. The production of OMVs is still unclear, and why all bacterial cargo can be detected in OMVs remains unknown. However, a growing body of biological evidence is being collected to explain these complex processes. The ability of OMVs to transport a significant amount of the biological materials present in the parent bacterium to distant sites in the host, thus facilitating bacterial communication, the transmission of virulence factors, and the maintenance of bacterial communities, is ultimately due to their capacity to carry cargo [44]. To date, although the mechanism of OMV production remains unclear, three models of OMV biogenesis have been proposed.

#### 2.2.1. Reduced Lipoprotein Linkages

Hypervesiculation is often observed in the case of reduced crosslinking between the outer membrane and the underlying peptidoglycan layer. The reduced number of linkages can lead to the bulging of the outer membrane, thus resulting in the production of vesicles. Dick Hoekstra already showed that vesicles may be easily formed in areas with a low number of outer membrane–murein linkages [45]. Similar results can also be found in the reports by Meta J. Kuehn and Maria Kaparakis-Liaskos [46,47]. Both *Acinetobacter baumannii* and *Escherichia coli* have similar OMV production tendencies with reduced crosslinking.

#### 2.2.2. Peptidoglycan Residues

According to this hypothesis, there are locations where the concentration of peptidoglycans is greater during the production of the peptidoglycan layer, which causes protrusions in the outer membrane, signaling the start of vesicle formation. OMVs are released when peptidoglycan residue accumulation causes the outer membrane to bulge even more. For example, Meta J. Kuehn et al. reported that periplasmic peptidoglycan components could increase OMV biogenesis [48].

#### 2.2.3. Electrically Charged Lipopolysaccharides (LPSs)

Electrically charged LPSs are the subject of the third and final OMV production model. Positively charged LPSs and neutrally charged LPSs are the two forms of LPSs that *Pseudomonas aeruginosa* produce. OMVs are predominantly made of negatively charged LPSs when produced by *Pseudomonas aeruginosa* under oxidative stress. Due to the repelling effect of the negatively charged outer membrane, an increase in negatively charged LPSs within the cell envelope might facilitate the release of OMVs [49].

#### 2.2.4. Other Mechanisms

OMV biogenesis appears to be a diverse multi-factorial process stimulated or regulated by a variety of pathways/mechanisms that may function simultaneously. For example, the environment has been found to have an important role in OMV production. Kuehn et al. reported that OMV production is a stress-responsive process and that temperature could have an effect on Serratia marcescens and *E.coli* [48,50]. Wael Elhenawy et al. reported in the *Salmonella enterica serovar Typhimurium* model that a lipid is necessary for OMV biogenesis [51], that is, deacylation happens within a multifactorial procedure, including planned modification of the outer membrane. Lynne Turnbull et al. used super-resolution microscopy to observe the OMV production process in *Pseudomonas aeruginosa* biofilms [52]. The release of cytosolic material in *Pseudomonas aeruginosa* biofilms was found to result from the explosive cell lysis of a subpopulation of cells. Explosive cell lysis also generates broken membrane fragments that form OMVs quickly. Sandro Roier et al. showed that VacJ/Yrb ABC (ATP-binding cassette) is also involved in OMV formation [53]. VacJ/Yrb ABC is a phospholipid transporter. The accumulation of phospholipids in the outer leaflet of the outer membrane causes this general mechanism of OMV generation.

## 3. Drug Delivery Performance of PVs and OMVs

Recently, it has been found that both PVs and OMVs can be employed in a variety of bio-applications, such as drug delivery [15,29,54,55,56], vaccination [57,58,59], cancer therapy [60,61,62], bioimaging [63,64], biosensing [65,66], and antibacterial applications [39,66,67,68,69,70,71,72]. PVs are generally obtained with amphiphilic block copolymers; thus, hydrophobic membranes can very easily carry hydrophobic drugs. In addition, delivering biomolecules from the parent bacterium to specific distant sites is one of the important functions of OMVs in nature. Further, the stimulus-responsive ability of PVs and OMVs offers the best way to release the loaded drug. Under specific stimuli, such as physical (heat or light), chemical (pH and reducing agents), and biomarker (e.g., ATP or RNA) agents, PVs and OMVs can also respond in a certain way to achieve targeted drug release. Both PVs and OMVs have natural advantages in terms of drug delivery (Table 2). 

### 3.1. Advantages of PVs and OMVs in DDSs

Due to multiple advantages, both PVs and OMVs can be used as platforms in DDSs [15]. The first advantage is the suitable size. PVs can be assembled in vesicles of suitable size by changing the nanoprecipitation conditions. The solvent and injection rate are the main impact factors when water is added to the polymer solvent. Furthermore, pH, polymer concentration, and temperature are important factors in the design of the size of vesicles. Moreover, the size can be altered from several nanometers to micrometers. OMVs commonly have sizes of 50 nm~250 nm. The size of both PVs and OMVs can allow them to pass through the tumor cell membrane owing to the enhanced permeability and retention effect [27]. Secondly, they are biocompatible and biodegradable. Due to the fact that they are derived from natural compounds, both PVs and OMVs are biocompatible and enzyme biodegradable. For example, Sofroniew and Deming showed that amphiphilic polypeptides can be used for the sustainable release of cargo; these polypeptides were detected to have no detectable toxicity to normal cells and could be fully degraded over several weeks [85,86]. OMVs have also been shown to be environmentally inert and biodegradable. Using electron microscopy to analyze OMV morphology, Schulz et al. also discovered that OMVs are biocompatible with differentiated macrophages and epithelial cells [87]. Thirdly, both PVs and OMVs can easily be loaded with the drug compound and protect it [88,89]. Fourthly, both PVs and OMVs can easily deliver drugs to target sites. For example, folic acid can be conjugated with a polypeptide chain and be used as the targeted group [9,90,91,92]. By genetically modifying the parent bacterium, targeting ligands can be added to OMVs. Targeting ligands make it easier for medications to build up at the desired sites. Lastly, PVs can be designed and conjugated with fluorescence groups, such as pyrene or NIR-II, which makes superior fluorescence imaging possible [93,94]. OMVs are produced from bacteria and contain a variety of pathogen-associated molecular patterns; because neutrophils and macrophages can recognize and internalize them, OMVs can also be employed to deliver specific cargoes to these cells [95,96].

Additionally, they each have their own advantages. For example, the chemical synthetic approaches designed so far allow polypeptide-based vesicles to be more easily designed. The stimulus properties of PVs can be engineered with various responsive groups. poly(N-isopropylacrylamide) (PNIPAM) has been found to undergo thermally responsive transformation in water at 32 °C [97]. After conjugating with PNIPAM, PVs become sensitive to temperature, thus achieving selective release. Furthermore, amino acids can be altered depending on the tumor environment. By selecting poly(L-histidine), pH-responsive vehicles were designed based on the closed pKa ≈ 6.0 of the tumor environment [98,99]. In addition, polypeptides can be obtained in just a few minutes depending on the development of the catalysts. Thus, PVs have a bright future in mass production. OMVs trigger an immunological response that is advantageous for the treatment of tumors. This, however, also presents disadvantages, as the immunological response might harm the host if proper measures are not taken to control it.

### 3.2. Techniques for Drug Loading in PVs and OMVs

#### 3.2.1. Drug Loading in PVs

**Figure 5:**
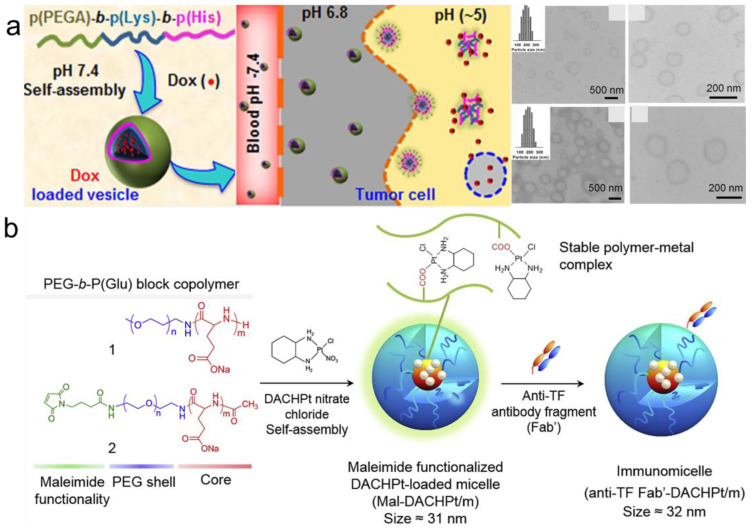
Drug loading in PVs. (**a**) p(PEGA)_30_-b-p(Lys)_25_-b-p(His)_n_ assembled into vesicles with DOX loading. Reprinted with permission from Ref. [74]. Copyright: 2015, American Chemical Society. (**b**) DACHPt loading into PEG-b-PLG copolymers. Reprinted with permission from Ref. [77]. Copyright: 2015, Elsevier.

In PV drug loading, drugs can be divided into hydrophobic drugs, hydrophilic drugs, and metal-based drugs [100,101]. Hydrophobic interaction is the main approach for loading hydrophobic drugs. For example, Sebastien Lecommandoux et al. reported that PEO-b-PBLG diblock and PBLG-b-PEO-b-PBLG triblock copolymers could be loaded with the hydrophobic drug doxorubicin (DOX) [73]. DOX could encapsulate as much as 18 wt.%. Kim et al. showed that poly(ethylene glycol) methyl ether acrylate-block-poly(l-lysine)-block-poly(l-histidine) [p(PEGA)_30_-b-p(Lys)_25_-b-p(His)_n_] can be used for the design of and assembly into the vesicles [74] (Figure 5a). These vesicles, with suitable sizes of 200~372 nm and hydrophobic chains of p(His), were used as a loading site for DOX. The content of DOX was as high as 21 ± 2% and the loading efficiency was 68%.

Besides hydrophobic drugs, hydrophilic drugs can be loaded into PVs through electrostatic interactions, and metallic coordination can easily encapsulate metal-based drugs. For instance, Chen’s group developed PEG-b-poly(l-glutamic acid) (PEG-b-PLG) through the ROP of NCAs using PEG-NH_2_ as the initiator [75]. Having a portable amino group in the sugar moiety, DOX·HCl is a positively charged and amphiphilic drug. The carboxylate of the glutamic acid units is then electrostatically loaded with DOX-HCl, creating mPEG-b-PLG-DOX·HCl through intermolecular hydrophobic stacking. Kazunori Kataoka’s group prepared oxaliplatin and (1,2-diaminocyclohexane)platinum(II) (DACHPt)-loaded PEG-b-PLG copolymers [77] (Figure 5b). This stable polymer metal complex could be formed to induce drug-loaded PVs.

#### 3.2.2. Drug Loading in OMVs

**Figure 6:**
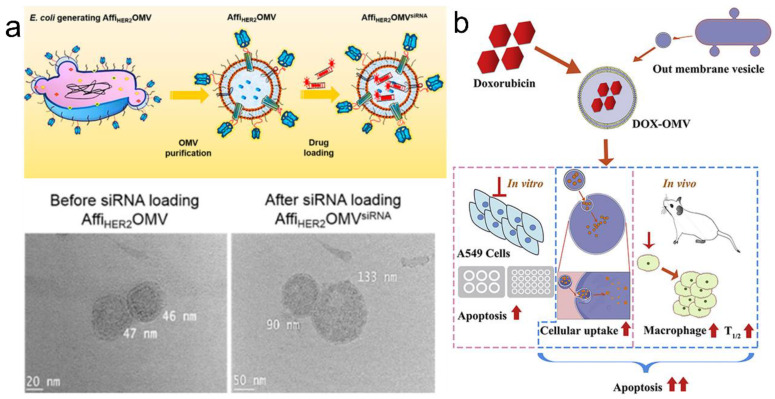
Drug loading in OMVs. (**a**) Loading of siRNA into Affi_HER2_OMVs. Reprinted with permission from Ref. [55]. Copyright: 2014, American Chemical Society. (**b**) DOX can be loaded into OMVs via passive diffusion. Reprinted with permission from Ref. [76]. Copyright: 2020, Elsevier.

The in vitro and in vivo loading methods are the two main techniques for drug loading in OMVs. The lipid bilayer of OMVs endows them with the potential to be loaded with both hydrophobic and hydrophilic drugs. For example, Sangyong Jon et al. showed that electroporation can be used for loading siRNA into Affi_HER2_OMVs [55] (Figure 6a). This method was well tolerated by OMVs as evidenced by the integrity of OMVs following siRNA loading and the preserved circular shape. Li Ye’s group also showed that DOX could be loaded into OMVs via passive diffusion [76] (Figure 6b). OMVs from *Klebsiella pneumonia* were loaded with DOX via passive diffusion in 12 h. DOX fluorescence was observed, which might have concentrated in the nucleus due to the preferential DNA-binding property of DOX. Besides these approaches, incubation, ultrasonication, and extrusion have been reported to be effective methods for in vitro drug loading.

The uniqueness of drug loading in OMVs is that the drug loading process can also be designed during their biogenesis. With such a bioengineering process, it is possible to load cancer treatment drugs. During the generation of OMVs, the incorporation of the drug compound within their parental bacterium is possible. Terry J. Beveridge’s group reported on the treatment of *Pseudomonas aeruginosa* PAO1 with gentamicin; the released OMVs were found to have the gentamicin [102]. Moreover, gentamicin effects that could arrest cancer cell growth were observed. Ana L. Carvalho et al. showed that OMVs could produce keratinocyte growth factor-2 (KGF-2), a human therapeutic protein, in a stable form [78]. It decreased disease severity when given orally and encouraged intestinal epithelium repair and recovery in animals given colitis-inducing dextran sodium sulfate. Vasilis Ntziachristos’ group also showed that the engineered *E. coli* could express rate-limiting enzymes in melanin biosynthesis [79]. Thus, the resulting OMVs could kill melanin in vivo. These reports suggest that OMVs could induce cytokine-mediated antitumor activity.

### 3.3. Targeted Drug Delivery via PVs and OMVs

#### 3.3.1. Targeted Drug Delivery via PVs

After loading the drug compound, its programmed delivery and release at the targeted site are significant. In PVs, changes in the polypeptide assembly behavior and drug–polypeptide interactions are the two main approaches to releasing the drug compound. Since PVs can be easily destroyed due to changes in pH, light, glucose, and reactive oxygen species (ROS) [104] and due to the acidic environment of the tumor, pH responsiveness is the most common stimulus used to release the drug compound. For instance, the sensitivity of poly(l-histidine) could allow the released DOX load to kill cancers [105]. Ding et al. reported that the micelle-to-vesicle transition could be observed on a cholesterol-decorated polypeptide [80]. Due to ROS changes, the conformation of the polypeptide could change from β-sheet to α-helix and further induce on-demand drug release at the targeted sites. The advantage of synthesized polypeptides is that the various environmentally sensitive nonpeptide groups can be conjugated on light polymer chains. Light- and glucose-responsive groups such as phenylboronic acid, spiropyran, and coumarin can change the assembly behavior upon environmental changes such as UV irradiation and insulin delivery systems. For instance, Dong’s group showed that it is possible to first design S-(o-nitrobenzyl)-l-cysteine and then block it with PEG via ROP [81]. The o-nitrobenzyl groups can be leveled off with UV irradiation. Moreover, the release of the drug compound can be controlled using the irradiation time. 

**Figure 7:**
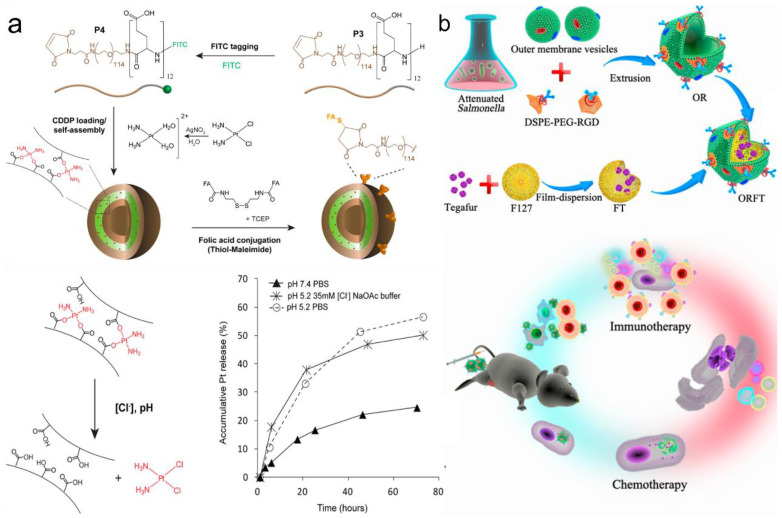
Targeted drug delivery via PVs and OMVs. (**a**) Drug release ability of cisplatin complexed with the PEG-b-PLG. Reprinted with permission from Ref. [103]. Copyright: 2015, American Chemical Society. (**b**) Schematic diagram of bioengineered OMV-coated polymeric nanomedicine. Reprinted with permission from Ref. [83]. Copyright: 2020, American Chemical Society.

Another important approach is destroying the interactions between the drug compound and the polypeptides. As mentioned above, the drug compound can be covalently or non-covalently conjugated with the polypeptides. Metal complexation and covalently linked drugs bearing polypeptides are mainly used in this release method. Kazunori Kataoka et al. designed hydrazide groups as the linkages between polypeptide chains and the drug DOX [82]. The hydrazide group can be disrupted as the pH changes and further release free DOX. Greg G. Qiao’s group showed that ligand exchange could be used for metal-based drug delivery [103] (Figure 7a). Cisplatin complexed with PEG-B-PLG could be released in the presence of chloride ions. However, it was found that the metal–ligand exchange could only lead to a relatively slow release of the drug compound. This release rate was enhanced when mimicking the endosomal/lysosomal environment (pH 5.2/35 mM [Cl^−^]).

#### 3.3.2. Targeted Drug Delivery via OMVs

Compared with the scalable and chemically designable PVs, the use of OMVs in targeted drug delivery has only been sporadically reported in recent years and is still an emerging area requiring urgent research [28]. Sangyong Jon’s study is recognized as the first report on using OMVs for targeted drug delivery [55]. After the electroporation of the siRNA drug compound (~15%) into OMVs, the anticancer activity of the drug-containing OMVs was found to be able to inhibit cancer cell proliferation. The detailed mechanism was observed with a confocal microscope as follows: the acidic environment could enhance the degradation of OMVs, and free siRNA was gradually detected along with dead cancer cells. Further, an in vivo study also suggested that the EPR effect is the drug force necessary for targeted drug delivery. Recently, Ping et al. showed that *Salmonella*-based OMVs could be even coated on polymer F127 nanoparticles [83] (Figure 7b). In this case, the drug compound was loaded on polymer micelles, whereas the OMVs only triggered the host immune response to cancer immunotherapy. This OMV-coated type of DDS provides a new combined system for potential targeted drug delivery. In addition, some recently emerged research also showed that bioengineered OMVs could induce specific CD8 T cell response, and this cell could control tumor growth via viral replication [84]. These recent findings open further possibilities for OMV drug delivery studies.

## 4. Conclusions and Outlook

This review mainly describes PVs and OMVs in drug delivery applications. PVs are obtained with the artificial synthesis of polypeptides, where the NMM and AMM are the two major mechanisms for polypeptide synthesis via the ROP of NCAs. Polymer vesicles are then obtained via self-assembly. The anisotropic packing of α-helical, hydrophobic polypeptides and the use of oppositely charged polymers are methods for the preparation of PVs. Compared with PVs, OMV biogenesis remains a mystery; however, three models, i.e., reduced lipoprotein linkages, peptidoglycan residues, and electrically charged lipopolysaccharides, are recognized as the major influencing factors. In addition, other factors, such as the environment and lipids, are important for OMV biogenesis in certain bacteria. Finally, the similarities and differences between these two vesicle types in drug delivery applications are elaborately compared. Specifically, we compare their applications in DDSs in the following aspects: general advantages, drug loading techniques, and targeted drug delivery performance. Based on state-of-the-art PVs and OMVs, we believe that future research should address the following issues to advance toward further DDS applications:

(1) Mass production of OMVs and PVs: Currently, the production of OMVs and PVs is still in the laboratory research stage. Although, recently, the production efficiency of polypeptide materials has been rapidly improved, technical solutions for assembling vesicles still lack specific standards, and large-scale production of PVs is not yet possible. OMV sizes are difficult to regulate, and there is significant variance among batches. In addition, low particle programmability is caused by our lack of knowledge regarding the precise OMV assembly mechanism. Enhancing safety and reducing costs are also still problems that need to be addressed.
(2) Universality of drug loading and delivery targeting: The drug loading mechanism in the process of production and post-treatment is inefficient and is in urgent need of optimization. Given the relevance of various cancer types and given that there are various PV and OMV types, consistent tumor cell targeting standards still need to be achieved and further improved. Individual PVs and OMVs may be effective for certain cancers.
(3) Other specific issues: The immunotoxicity of OMVs is a problem that cannot be ignored in DDSs. Moreover, the artificial production of OMVs also deserves more in-depth study on its potential. In PVs, the deprotection process in the production of specific polypeptides (such as polyhistidine, polylysine, and polycysteine) is tedious. Finally, programmed drug release and the benefits of the unique secondary structure of PVs are also worthy of more in-depth research.

## Figures and Tables

**Table 1:** Comparison of characteristics of PVs and OMVs.

Classification	PVs	OMVs	Reference
Origin	Synthetic polymers	Gram-negative bacteria	[13,17]
Production	Solution self-assembly	Biogenesis	[15,21]
Size	Several nanometers~micrometers	20~300 nm	[14,27]
Biomedical applications	Antibacterial	Bacterial vaccines	[14,28]
	Mineralization	Adjuvants	
	Drug delivery	Cancer immunotherapy	
	Imaging and sensing	Drug delivery	
	Therapeutic	Anti-bacteria adhesion	

**Table 2:** Comparison of drug loading and releasing by PVs and OMVs.

DDS	PVs	References	OMVs	References
Drug loading	Hydrophobic interaction	[73,74]	Electroporation	[55]
	Electrostatic interactions	[75]	Passive diffusion	[76]
	Metal coordination	[77]	Biogenesis	[78,79]
Drug releasing	Change peptide assembly behavior	[80,81]	Degradation of OMVs	[55]
	Change drug–polypeptide interaction	[82]	Coating on polymer	[83]
			Bioengineering	[84]

## Data Availability

Data supporting this publication are available from the corresponding authors.
